# Supplementary material for: Differential contributions of the two cerebral hemispheres to temporal and spectral speech feedback control
Source: Nat Commun. 2020 Jun 5;11:2839. doi: 10.1038/s41467-020-16743-2 (PMC7275068; doi:10.1038/s41467-020-16743-2)
Supplement: Supplementary file 1 — Supplementary Information [file 41467_2020_16743_MOESM1_ESM.pdf]

Supplementary Information

for

Differential contributions of the two cerebral hemispheres to temporal and spectral speech feedback control

Floegel et al

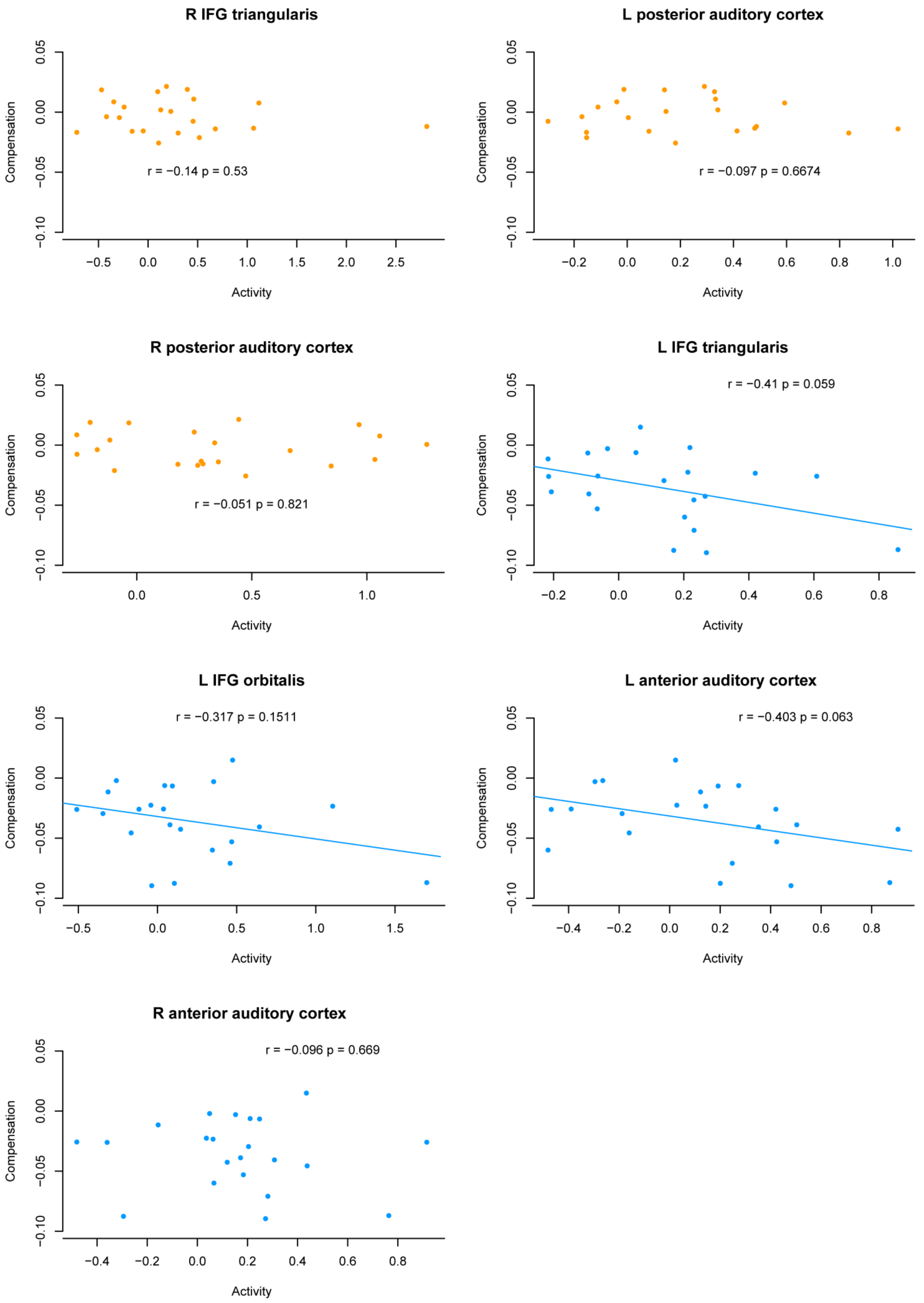

Supplementary Figure 1. Association between activity in feedback control related areas and the individual degree of compensation. The scatterplot shows individual datapoints for speaking with spectrally (orange) or temporally (blue) altered auditory feedback. Trends are indicated with a regression line ($0.05 < p < 0.06$, two-sided t-test of the pearson's correlation coefficients uncorrected for multiple comparisons, $n = 22$ participants per group).

Supplementary Table 1. Activation loci from Niziolek and Guenther, 2013 for ROI definition[1]. Coordinates are given in MNI space.

ROI allocation	x	y	z
Auditory	+/- 51	-15	-6
Auditory	+/- 55	-3	-13
Auditory	+/- 55	-32	0
Auditory	+/- 55	-20	0
Auditory	+/- 63	-45	10
Frontal	+/- 52	30	5
Frontal	+/- 33	30	-14
Frontal	+/- 40	18	4

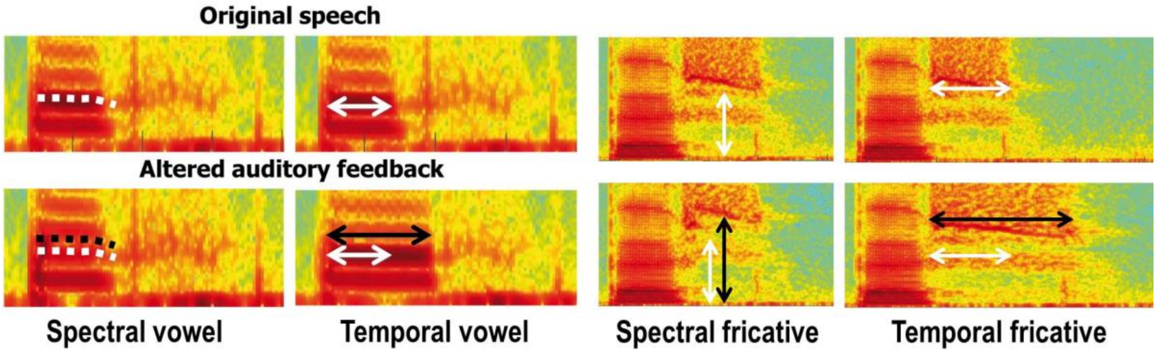

Supplementary Figure 2. Exemplary illustration of experimental feedback perturbations (single trial). The spectrograms shows the CVC-pseudoword bisch before (upper panel) and after (lower panel) spectral and temporal alteration of the vowel or fricative in the utterance. White arrows/points denote the speech feature before manipulation. Black arrows/points after feedback perturbation.

Supplementary References

1. Niziolek, C.A. & Guenther, F.H. Vowel category boundaries enhance cortical and behavioral responses to speech feedback alterations. *The Journal of Neuroscience* **33**, 12090–12098 (2013).
