# Supplementary material for: Differential contributions of the two cerebral hemispheres to temporal and spectral speech feedback control
Source: Nat Commun. 2020 Jun 5;11:2839. doi: 10.1038/s41467-020-16743-2 (PMC7275068; doi:10.1038/s41467-020-16743-2)
Supplement: Supplementary file 3 — Reporting Summary [file 41467_2020_16743_MOESM3_ESM.pdf]

nature research

Corresponding author(s): Christian A. Kell

Last updated by author(s): May 12, 2020

Reporting Summary

Nature Research wishes to improve the reproducibility of the work that we publish. This form provides structure for consistency and transparency in reporting. For further information on Nature Research policies, seeAuthors & Referees and theEditorial Policy Checklist .

Statistics

For all statistical analyses, confirm that the following items are present in the figure legend, table legend, main text, or Methods section.

n/a	Confirmed	
☐	☒	The exact sample size (n) for each experimental group/condition, given as a discrete number and unit of measurement
☐	☒	A statement on whether measurements were taken from distinct samples or whether the same sample was measured repeatedly
☐	☒	The statistical test(s) used AND whether they are one- or two-sided<br>*Only common tests should be described solely by name; describe more complex techniques in the Methods section.*
☒	☐	A description of all covariates tested
☐	☒	A description of any assumptions or corrections, such as tests of normality and adjustment for multiple comparisons
☐	☒	A full description of the statistical parameters including central tendency (e.g. means) or other basic estimates (e.g. regression coefficient) AND variation (e.g. standard deviation) or associated estimates of uncertainty (e.g. confidence intervals)
☐	☒	For null hypothesis testing, the test statistic (e.g. F, t, r) with confidence intervals, effect sizes, degrees of freedom and P value noted<br>*Give P values as exact values whenever suitable.*
☒	☐	For Bayesian analysis, information on the choice of priors and Markov chain Monte Carlo settings
☒	☐	For hierarchical and complex designs, identification of the appropriate level for tests and full reporting of outcomes
☐	☒	Estimates of effect sizes (e.g. Cohen's d, Pearson's r), indicating how they were calculated

Our web collection on statistics for biologists contains articles on many of the points above.

Software and code

Policy information about availability of computer code

Data collection	Matlab 2012 and Audapter: Stimulus presentation and sound recording
Data analysis	PRAAT: Manual labeling of recorded speech files. Extraction of speech features<br>R 3.5.3: Statistical analysis of behavioral data and visualization<br>Matlab 2012: Environment for fMRI analysis<br>SPM 12: Analysis of fMRI task activation<br>Conn toolbox 17f: functional connectivity resting state analysis<br>LI toolbox: Calculation weighted lateralization indices of functional imaging contrasts<br>Art Repair v4: artifact detection, motion correction of functional data<br>MRIcron 2016: visualization of fMRI data

For manuscripts utilizing custom algorithms or software that are central to the research but not yet described in published literature, software must be made available to editors/reviewers. We strongly encourage code deposition in a community repository (e.g. GitHub). See the Nature Research guidelines for submitting code & software for further information.

Data

Policy information about availability of data

All manuscripts must include a data availability statement. This statement should provide the following information, where applicable:

- Accession codes, unique identifiers, or web links for publicly available datasets
- A list of figures that have associated raw data
- A description of any restrictions on data availability

Unthresholded statistical parametric maps are available at https://identifiers.org/neurovault.collection:7569. The source data underlying Figure 1, 2, 3a, 3c and supplementary Figure 1 are provided as a source data file.

Field-specific reporting

Please select the one below that is the best fit for your research. If you are not sure, read the appropriate sections before making your selection.

☒ Life sciences ☐ Behavioural & social sciences ☐ Ecological, evolutionary & environmental sciences

For a reference copy of the document with all sections, see nature.com/documents/nr-reporting-summary-flat.pdf

Life sciences study design

All studies must disclose on these points even when the disclosure is negative.

Sample size	Sample sizes were based on previous published studies that reported significant effects for speaking with altered auditory feedback compared to normal speaking with sample sizes below 20 (e.g. Tourville et al. 2008 in = 11 in a restricted search space and Niziolek & Guenther 2013 n = 18 whole brain analysis). We therefore used n = 20 for our groups.
Data exclusions	Data was excluded if the real-time tracking algorithm did not consistently work throughout the experiment (Franken et al. 2019. Exp Psychology, Vol. 72, van den Bunt 2018 Scientific studies of reading, Vol. 22) or participants did not follow task instructions (e.g. singing instead of speaking).
Replication	Data regarding lateralization was analyzed from a behavioral study and an fMRI study and yielded consistent effects. Further, fMRI data was analyzed in different ways to assess lateralization (weighted LIs, direct statistical comparison) and yielded complementary results.
Randomization	Participants were randomly allocated to experimental groups
Blinding	The investigators were not blinded with respect to group allocation. However, the relevant measures which may be influenced by knowledge about task conditions (i.e. changes in produced phonem length) were labelled by a blinded annotator

Reporting for specific materials, systems and methods

We require information from authors about some types of materials, experimental systems and methods used in many studies. Here, indicate whether each material, system or method listed is relevant to your study. If you are not sure if a list item applies to your research, read the appropriate section before selecting a response.

Materials & experimental systems

n/a	Involved in the study	
☒	☐	Antibodies
☒	☐	Eukaryotic cell lines
☒	☐	Palaeontology
☒	☐	Animals and other organisms
☐	☒	Human research participants
☒	☐	Clinical data

Methods

n/a	Involved in the study	
☒	☐	ChIP-seq
☒	☐	Flow cytometry
☐	☒	MRI-based neuroimaging

Human research participants

Policy information about studies involving human research participants

Population characteristics	40 healthy volunteers (20 female) participated in the behavioral experiment and 44 healthy volunteers (27 female) in the fMRI study. Participants were adult native right-handed speakers of German [handedness score behavioral study 93 (9.8), fMRI study 90 (11.5)] and reported normal speech and hearing.
Recruitment	Flyer at university. Advertisement in student facebook groups. This recruitment method biased participants to students at Goethe univeristy Frankfurt. The rather young age of our sample (18-35) may restrict generalization of findings to older participants
Ethics oversight	The study was approved by the ethics committee of the Medical Faculty of Goethe-University Frankfurt (DFGKE 1514/2-1) and was in accordance with the Declaration of Helsinki.

Note that full information on the approval of the study protocol must also be provided in the manuscript.

Magnetic resonance imaging

Experimental design

Design type	Event-related, sparse sampling design; Continuous resting state.
Design specifications	Task fMRI: 2 Conditions a 30 trials + control condition with 30 occurences<br><br>Each trial started with the 2 seconds long acquisition of one functional image. Image acquisition was followed by a pause of 0.5-1.5s after which the CVC pseudoword or the non-speech stimulus was visually presented for 2 seconds. After another pause of 2.5-3.5 seconds, the next image was acquired resulting in a total trial length of 8 seconds.<br>Resting_state: Two 7min runs. One before and one after speech adaptation.
Behavioral performance measures	Audio recording of participants utterance.<br>Extraction of F1, COG vowel and fricative lengthes. LMMs to asses whether participants changed their production.

Acquisition

Imaging type(s)	functional, structural
Field strength	3
Sequence & imaging parameters	High-resolution T1-weighted anatomical scans [TR = 1.9s; TE =3.04ms; flip angle = 9°; 192 slices per slab; 1mm3 isotropic voxel size]<br><br>Functional images were obtained with a gradient-echo T2*-weighted transverse echoplanar image (EPI) sequence [Task-fMRI (122 volumes; TR = 2s; TE = 30ms; silent gap = 6s; flip angle = 90°; 32 axial slices; 3mm3 isotropic voxel size), Resting-State (178 volumes; TR = 2s; TE = 30ms; flip angle = 90°; 30 axial slices; 3mm3 isotropic voxel size)].
Area of acquisition	whole brain

Diffusion MRI ☐ Used ☒ Not used

Preprocessing

Preprocessing software	standard SPM 12 spatial preprocessing pipeline + ArtRepair motion correction (Realignment of functional images using rigid body transformation, co-registration, smoothing of images with an isotropic 4mm full-width at half-maximum Gaussian kernel to prepare images for additional motion adjustment with Art Repair, motion adjustment of functional images with ArtRepair to reduce interpolation errors from the realignment step, normalization of functional images to a symmetric brain template via parameters from segmentation of structural scans, final smoothing of images with an isotropic 7 mm full-width at half-maximum Gaussian kernel.<br><br>Additional denoising steps for resting state data are described below.
Normalization	Normalization via segmentation
Normalization template	Data was normalized with a symmetric version of the standard Montreal Neurological Institute (MNI) brain template within the Talairach and Tournoux reference frame. The symmetric version was created by averaging the original version with its R/L flipped version.
Noise and artifact removal	Motion correction using rigid body transformation.<br><br>Resting state data was further denoised to reduce the impact of physiological noise and motion on results. Physiological noise was removed with the anatomical component-based noise correction method (aCompCor) and 16 orthogonal time-courses in subject-specific WM and CSF ROIs. Further, subject-specific motion parameters and their first derivative (scan-to-scan motion), task-effects and subject-specific time points identified as outliers (scan-to-scan global signal change > 9 and movement more than 2 mm) were regressed out. To isolate low frequency fluctuations, resting-state data were bandpass filtered (0.008-0.09 Hz)
Volume censoring	scan-to-scan global signal change > 9 and movement more than 2 mm

Statistical modeling & inference

Model type and settings	Mass univariate approach<br>Task<br>The GLM contained 3 regressors of interest, modelling the three auditory feedback conditions (no perturbation, vowel perturbation, consonant perturbation). Due to the additional motion adjustment step during preprocessing movement-related effects were not modelled additionally. Condition-specific regressors were obtained by convoluting the onset and duration of conditions (modelled by boxcar functions) with the canonical hemodynamic response function. To account for the use of a sparse sampling protocol, we adjusted microtime resolution and onset (SPM.T = 64, SPM.T0 =

8s). The model was high pass filtered with a cut off at 128s to remove low frequency drifts. An autoregressive model AR (1) was used to account for serial autocorrelations in the time series.
After model estimation, two contrasts were specified testing the effect of speaking with altered auditory feedback (vowel or consonant perturbation) against normal speaking without perturbation in each individual (first-level). The resulting contrast images were subjected to second level random effect analysis to infer brain activation at the population level.

Resting State
For each participant and each resting-state run (pre- and post adaptation) seed-to-voxel connectivity maps were generated by calculating bivariate correlations between the average seed time-series and the whole brain (seeds: 6mm spheres centered on local peak activation in spectral/temporal feedback control contrasts). The second level GLM contained two regressors representing changes in connectivity between resting state runs (one for the spectral group and another for the temporal group) and 4 parametric regressors that represented the subject-specific amount of compensation for spectral or temporal perturbations of the vowel and consonant, separately.

Effect(s) tested

Task fMRI:
Differences in activity and lateralization between speaking with spectrally/temporally altered speech feedback and normal speaking (conjunction analysis over vowel and consonant regressors)
Correlations between the individual degree of compensation and brain activity in feedback-control related brain areas.

Resting State: motor learning-related connectivity changes by means of conjunction analyses over vowel and consonant regressors (e.g. post/pre adaptation difference that correlates with F1 compensation ∩ post/pre adaptation difference that correlates with COG compensation).

Specify type of analysis: ☐ Whole brain ☐ ROI-based ☒ Both

Anatomical location(s): SPM anatomy toolbox (probabilistic atlas)

Statistic type for inference (See Eklund et al. 2016): FWE correction at the cluster level with a cluster defining threshold of 0.001

Correction: FWE

Models & analysis

n/a	Involved in the study
☐	☒ Functional and/or effective connectivity
☒	☐ Graph analysis
☒	☐ Multivariate modeling or predictive analysis

Functional and/or effective connectivity: Pearson correlation between average time series in a seed and the whole brain
